# Assessment of the 1,3-β-D-glucan test and the galactomannan antigen test in the detection of invasive fungal infections in patients with hematological diseases

**DOI:** 10.1128/spectrum.01209-25

**Published:** 2025-09-03

**Authors:** Zhaohui Wei, Shuguang Li, Jiabao Zhu, Hui Wang

**Affiliations:** 1 Institute of Medical Technology, Peking University Health Science Center 33133, Beijing, China; 2 Department of Clinical Laboratory, Peking University People's Hospital 71185, Beijing, China; Jagiellonian University, Krakow, Poland

**Keywords:** 1,3-β-D-glucan test, galactomannan antigen test, invasive fungal infections, hematological diseases, sensitivity and specificity, *Candida*, *Aspergillus*

## Abstract

To evaluate the diagnostic performance of the 1,3-β-D-glucan (BDG) test and the galactomannan (GM) antigen test in invasive fungal infections (IFI) among hematologic patients and to explore an optimized diagnostic approach. A total of 294 consecutive patients with hematological diseases suspected of fungal infections were enrolled at Peking University People’s Hospital from January 2020 to October 2023. IFI diagnosis referred to a composite reference standard (CRS), which combined both mycological and clinical evidence. Serum BDG (cut-off: 60 pg/mL) and GM (cut-off: 0.5) tests were analyzed. Of the 294 patients, 122 were classified as IFI-positive, including cases of invasive Aspergillosis (IA, *n* = 55), invasive Candidiasis (IC, *n* = 49), and other fungal infections (*n* = 18). Compared with CRS, the BDG test demonstrated higher sensitivity (64.4% vs 47.5%, *P* = 0.008), while the GM test showed higher specificity (93.0% vs 83.1%, *P* = 0.003). The combination of BDG and GM test results generated a Predicted Diagnostic Probability Value (PDPV) and yielded a receiver operating characteristic (ROC) curve with an area under the curve (AUC) of 0.848. At a PDPV threshold of 0.64, the Youden index was maximized (*J* = 0.60), achieving 73.0% sensitivity and 86.6% specificity. PDPV outperformed both the BDG (*P* = 0.010) and GM tests (*P* = 0.002, *Z* test). In hematologic patients, the composite diagnostic indicator PDPV significantly improved diagnostic performance for IFI, offering higher sensitivity and robust specificity compared to the individual BDG or GM tests. Additionally, the BDG test detected significantly more positive IC cases than the GM test, though no significant difference was observed for IA diagnosis.

**IMPORTANCE:**

Invasive fungal infections (IFI) remain a common cause of morbidity and mortality in patients with hematologic diseases, and early diagnosis is crucial for improving outcomes. This study systematically evaluated the diagnostic performance of the 1,3-β-D-glucan (BDG) test and the galactomannan (GM) antigen test in a cohort of hematologic patients. Importantly, the study developed a novel composite indicator, the Predicted Diagnostic Probability Value (PDPV), which integrated BDG and GM results to enhance diagnostic performance. PDPV showed improved sensitivity and specificity compared to individual tests, offering a practical and optimized approach for IFI diagnosis in high-risk patients. This diagnostic approach may contribute to improved clinical decisions and earlier initiation of antifungal therapy.

## INTRODUCTION

Invasive Fungal Infections (IFI) can cause severe deep mycosis in humans. These fungi breach the body’s natural defense barriers, invading tissues, organs, or the circulatory system, potentially affecting patient health. Such infections typically occur in individuals with compromised immune systems or immunodeficiency, such as cancer patients, organ transplant recipients, and those on long-term immunosuppressive therapy (1). Numerous studies have confirmed that diabetes, respiratory diseases, neutropenia, liver and kidney failure, mucosal damage, and Epstein-Barr virus (EBV) infection are among the risk factors that increase a patient’s susceptibility to fungal infections (2–6). These conditions may act alone or in combination, diminishing the body’s ability to resist fungal invasions. In the field of blood disease treatment, hematopoietic stem cell transplantation (HSCT) is widely regarded as one of the effective treatments for various hematologic disorders. However, the chemotherapy and radiotherapy during the HSCT process, as well as the post-transplant immunocompromised state, significantly increase the risk of IFI in patients.

IFI is a serious medical issue. Therefore, timely and accurate diagnosis of IFI is crucial for improving patient outcomes. Although traditional microbial culture methods are considered the gold standard for diagnosis, their low sensitivity and long turnaround time limit their clinical effectiveness. 1,3-β-D-glucan (BDG) and galactomannan (GM) serve as serum biomarkers aiding in the adjunctive diagnosis of IFI and have been widely recognized and mentioned in various diagnostic guidelines (7–10). However, the BDG test and the GM test exhibit certain methodological instabilities and may yield divergent results when diagnosing different pathogens (11), and their sensitivity and specificity are often unstable; for instance, GM is more specific to invasive aspergillosis (IA), whereas BDG shows better sensitivity in detecting invasive candidiasis (IC).

Consequently, this study was designed to evaluate the performance of BDG and GM tests in diagnosing different types of IFI in patients with hematologic diseases, as well as to analyze the underlying factors that could influence the outcomes of these diagnostic tests, in order to provide more precise diagnostic criteria in clinical practice.

## MATERIALS AND METHODS

### Study population

The medical records of hematologic patients with suspected IFI were retrospectively collected in Peking University People’s Hospital from January 2020 to October 2023. Trained infectious disease specialists utilized a standardized electronic case report form (eCRF) to extract the following data: baseline demographical characteristics, comorbidities, hematological diagnosis, clinical features, laboratory results, anti-fungal treatment, and prognosis. To ensure data accuracy, two independent researchers reviewed all records. In cases of disagreement, a third reviewer was consulted to make the final decision.

The study employed a retrospective observational research design, combining both qualitative and quantitative analyses. The results of the most recent BDG test and serum GM test value conducted within 1 week (-7 days to 0 days) prior to the positive fungal culture were extracted for analysis. For the BDG test, Fungus (1-3)-β-D-Glucan Test (Chromogenic Method) (Beijing Jinshanchuan Technology Development Co., Ltd., China) was employed. For the GM test, Platelia Aspergillus EIA (Bio-Rad Laboratories, USA) was employed. According to the manufacturer’s instructions, the cut-off values for the BDG and GM tests were defined as 60 pg/mL and 0.5, respectively. These tests were part of the routine clinical assessment, providing references and warnings to assist in diagnosis, and the results were available to treating physicians during patient care.

This study adhered to the ethical policies mentioned on the journal’s author guidelines page and received approval from the Ethics Committee of Peking University People’s Hospital (No. 2024PHB249-001). The requirement for informed consent was waived as data were collected retrospectively and anonymized.

### Inclusion and exclusion criteria

Inclusion criteria were (1) hematologic disease with a history of HSCT or chemotherapy, and (2) patients with positive fungal culture results, in whom fungal species were identified by matrix-assisted laser desorption/ionization time-of-flight mass spectrometry (MALDI-TOF MS) and/or molecular methods. Patients missing results for either BDG or GM tests within one week before a positive fungal culture were excluded.

### Grouping criteria

The diagnostic criteria of IFI were considered comprehensively according to a composite reference standard (CRS), which combined both mycological and clinical evidence, mainly referring to the guidelines of the 2020 European Organization for Research and Treatment of Cancer and the Mycoses Study Group Education and Research Consortium (EORTC/MSGERC) and the Chinese guidelines for the diagnosis and treatment of invasive fungal disease (IFD) in patients with hematological disorders and cancers (the 6th revision) (7, 12). Briefly, its contents are as follows:

Based on the sample types and culture results, patients meeting the mycological evidence classified as “proven” or “probable” are classified into the IFI group (*n* = 110), including (i) cultivation of molds or black yeast-like fungi from sample obtained through aseptic techniques from normal sterile sites of the human body, excluding bronchoalveolar lavage fluid (BALF), cranial sinuses, and urine; (ii) cultivation of yeast from sterile specimens (including drainage fluid within 24 h); (iii) cultivation of molds (excluding *Aspergillus*), yeast, or yeast-like fungi from blood cultures; (iv) detection of fungal elements such as molds in sputum, BALF, bronchial brushings, or sinus aspirates, either through culture or suggestive growth of molds; (v) positive culture for novel *Cryptococcus* species from sputum or BALF, or the detection of *Cryptococcus* through direct microscopy or cytology; (vi) if the site of fungal isolation did not meet the predefined criteria for direct classification into the IFI group, patients with a clear clinical diagnosis indicating IFI (proven or probable) should still be classified into the IFI group (*n* = 12). Subsequently, based on culture results and clinical diagnoses, subgroups of IA, IC, and other IFI were further divided.

Patients who failed to meet the mycological criteria and had no sufficient clinical evidence (without receiving any antifungal treatment throughout the disease course) were classified into the non-IFI group.

### Statistical methods

Statistical analyses were performed using IBM SPSS Statistics (Version 25.0, IBM Corp., Armonk, NY, USA) for categorical and continuous data analyses, and MedCalc Statistical Software (Version 19.2, MedCalc Software Ltd, Ostend, Belgium) for receiver operating characteristic (ROC) curve analysis. Graphs were generated using GraphPad Prism (Version 10.0, GraphPad Software, San Diego, CA, USA).

Categorical data were presented as numbers accompanied by percentages and analyzed using the Chi-Square test and Fisher’s exact test. Continuous data were displayed as mean with standard deviation and assessed using independent samples *t*-test for two groups, and one-way ANOVA for more than two groups. For continuous data that do not follow a normal distribution, using the Mann-Whitney *U* test for two groups and the Kruskal-Wallis *H* test for multiple groups.

ROC curve analysis was employed to derive sensitivity, specificity, and the area under the curve (AUC). Differences between AUCs were assessed using the DeLong’s test (*Z* test) (13), which evaluates whether the difference between two correlated ROC curves is statistically significant. All tests were considered statistically significant at *P* < 0.05.

### Construction and internal validation of the predicted diagnostic probability value model

A binary logistic regression model was constructed using BDG and GM test values as independent variables to estimate the probability of IFI. The dependent variable was infection status, coded as 1 (infection) and 0 (non-infection). The predicted probability derived from this logistic regression model was referred to as the Predicted Diagnostic Probability Value (PDPV) and was used as a combined diagnostic indicator.

To evaluate the internal robustness of the model, a bootstrap validation procedure was performed using the boot and pROC packages in R (version 4.4.2, R Foundation for Statistical Computing, Vienna, Austria). The logistic regression model was constructed in SPSS based on BDG and GM values, and the PDPV were extracted. Using 1,000 bootstrap resamples, the AUC was computed for each iteration to assess the variability and stability of the model performance.

## RESULTS

### Baseline characteristics

**Fig 1:**
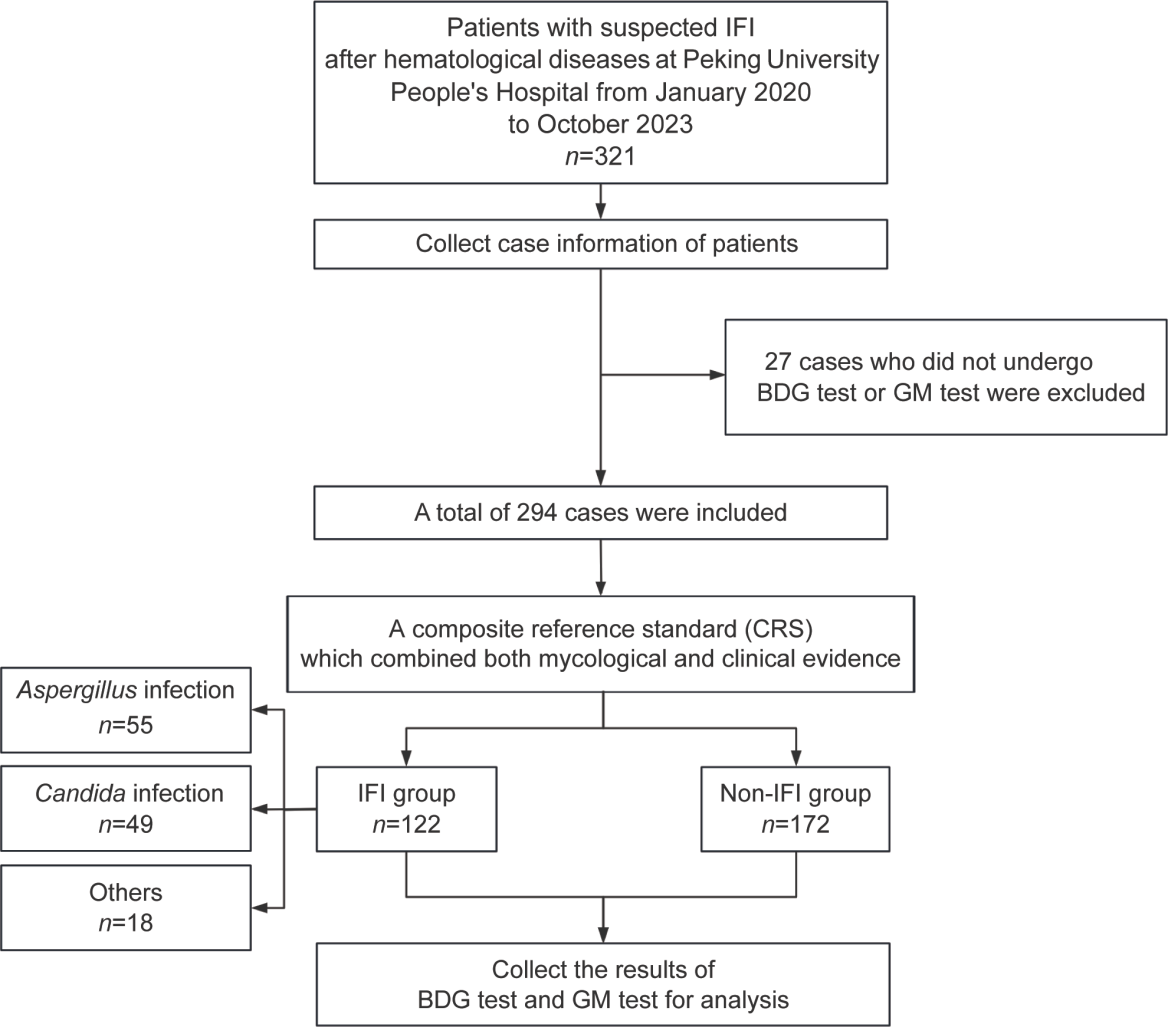
Flowchart of patient enrollment and classification of the study. The study included patients with hematologic diseases who were highly suspected of having IFI and were treated at Peking University People’s Hospital between January 2020 and October 2023 (*n* = 321). Focusing on results from the BDG test and GM test, a total of 294 cases were ultimately included. Based on microbiological evidence and clinical diagnosis, patients were classified into IFI (*n* = 122) and non-IFI groups (*n* = 172). The IFI group was further subdivided into *Aspergillus* infection (*n* = 55), *Candida* infection (*n* = 49), and other fungal infections (*n* = 18). BDG and GM test results, along with other clinical data, were collected for all groups for analysis. IFI, invasive fungal infections; BDG test, 1,3-β-D-glucan test; GM test, galactomannan antigen test.

Of the 321 patients initially enrolled in the study, 294 were included in the final analysis after excluding 27 patients who lacked either BDG test or GM test results. The cohort had a mean age of 52.3 ± 1.0 years, comprising 179 males (60.9%). Based on diagnostic classification criteria, 122 patients were categorized into IFI group, including 55 cases of IA, 49 cases of IC, and 18 cases of other fungal infections, while 172 patients were classified as non-IFI cases. Notably, the IFI group showed significantly higher proportion of patients who underwent HSCT compared to the non-IFI group (72.1% vs 42.4%, *P <* 0.001) (Fig. 1; Table 1).

**TABLE 1:** Characteristics of IFI and non-IFI group^*c*^

Characteristics	IFI group (*n* = 122)	Non-IFI group (*n* = 172)	*P* value
Age and mean (Std. Error)	47.0 (1.5)	56.1 (1.3)	0.221
Children	10.3 (1.6)	14.2 (1.1)	0.591
Adults	48.2 (1.5)	57.3 (1.2)	0.116
Children: Adults, *n* (%)	4 (3.3) :118 (96.7)	5 (2.9):167 (97.1)	1.000
Male: female, *n* (%)	75 (61.5): 47 (38.5)	104 (60.5):68 (39.5)	0.419
HSCT, *n* (%)	88 (72.1)	73 (42.4)	<0.001^*a*^
Underlying disease, *n* (%)			
AML	55 (45.1)	64 (37.2)	0.175
ALL	18 (14.8)	21 (12.2)	0.526
CML	4 (3.3)	1 (0.6)	0.192
MDS	12(9.8)	7 (4.1)	0.048^*a*^
AA	7 (5.7)	3 (1.7)	0.125
MM	9 (7.4)	39 (22.7)	<0.001^*a*^
NHL	8 (6.6)	26 (15.1)	0.024^*a*^
Others	9 (7.4)	10 (5.8)	0.591
History of diseases^*b*^, *n* (%)			
Fungal infection history	31 (25.4)	4 (2.3)	<0.001^*a*^
Respiratory disease	80 (65.6)	87 (50.6)	0.011^*a*^
Diabetes	17 (13.9)	30 (17.4)	0.419
Gastrointestinal mucosal injury	25 (20.5)	19 (11.0)	0.025^*a*^
Renal insufficiency	22 (18.0)	30 (17.4)	0.896
Liver dysfunction	62 (50.8)	63 (36.6)	0.015^*a*^
Viral infection	40 (32.8)	48 (27.9)	0.368
Bacterial infection	79 (64.8)	137 (79.7)	0.004^*a*^

^*a*^ *P* < 0.05.

^*b*^ Cover the time from the initiation of the first HSCT or chemotherapy to the occurrence of the current infection.

^*c*^ IFI, invasive fungal infections; HSCT, hematopoietic stem cell transplantation; AML, acute myeloid leukemia; ALL, acute lymphoblastic leukemia; CML, chronic myeloid leukemia; MDS, myelodysplastic syndromes; AA, aplastic anemia; MM, multiple myeloma; NHL, non-Hodgkin lymphoma.

In terms of disease history, IFI group and non-IFI group showed statistical differences (*P* < 0.05 for all) in fungal infections (25.4% vs 2.3%), respiratory diseases (65.6% vs 50.6%), gastrointestinal mucosal damage (20.5% vs 11.1%), liver dysfunction (50.8% vs 36.6%), and bacterial infection (64.8% vs 79.7%) (Table 1).

Regarding antifungal treatment, azoles were the most commonly used agents across both groups, with an overall use rate of 76.5%. Notably, the IFI group showed significantly higher use of triazoles (95.1% vs 63.4%), caspofungin (72.1% vs 37.8%), and amphotericin B (24.6% vs 9.3%) compared to the non-IFI group (*P* < 0.05 for all). Furthermore, 1-year survival data were collected as of 31 January 2024. The overall 1-year mortality was significantly higher in the IFI group than in the non-IFI group (*P* = 0.003), whereas no significant difference was observed in 30-day survival (Table S1).

### Sample sources and pathogenic characteristics

According to culture results, IFI were primarily caused by genera of *Aspergillus* and *Candida* (Table 2). Among the 122 patients of the IFI group, *Aspergillus* were detected in 55 cases (45.1%), with *Aspergillus fumigatus* (*A. fumigatus*) being the most common species, accounting for 19 cases (15.6%). Co-infections with *Aspergillus* species were found in 16 cases (13.1%), each co-infection has independent culture results. *Candida* species were detected in 49 cases (40.1%), with *Candida tropicalis* being the most frequently identified species, totaling 26 cases (21.3%). Co-infections with *Candida* species were observed in 4 cases (3.3%). Other pathogens, such as *Mucor* and *Fusarium*, were responsible for infections in 18 cases (14.8%) (Table 2).

**TABLE 2:** Pathogen culture result of IFI group

Pathogen culture result	*n* (%)
*Aspergillus*	55 (45.1)
*A. fumigatus*	19 (15.6)
*A. flavus*	8 (6.6)
*A. niger*	6 (4.9)
Other *Aspergillus*^*a*^	6 (4.9)
*Aspergillus*-associated co-infections^*b*^	16 (13.1)
*Candida*	49 (40.1)
*C. tropicalis*	26 (21.3)
*C. parapsilosis*	8 (6.6)
*C. albicans*	7 (5.7)
*C. glabrata*	4 (3.3)
*Candida*-associated co-infections^*c*^	4 (3.3)
Others^*d*^	18 (14.8)
Total	122 (100.0)

^*a*^ Other *Aspergillus* species include *A. versicolor* (*n* = 2), *A. terreus* (*n* = 2), unclassified *Aspergillus* (*n* = 2).

^*b*^ *Aspergillus*-associated co-infections include *A. fumigatus* and* A. flavus* (*n* = 4)*; A. fumigates, A. flavus*, and* A. niger* (*n* = 2); co-infections of* Aspergillus* (*n* = 6); and* Aspergillus* with one or two filamentous fungi (including* Aspergillus fumigates*, *n* = 1, non-*fumigates Aspergillus*, *n* = 3).

^*c*^ *Candida*-associated co-infections include *C. albicans* and *C. glabrata* (*n* = 1); and co-infections of *Candida* with one filamentous fungus (including *Candida albicans*, *n* = 2 and *Candida tropicalis, n* = 1).

^*d*^ Other types include *Fusarium* (*n* = 4); *Cunninghamella* (*n* = 2); *Rhizopus* (*n* = 2); *Trichosporon asahii* (*n* = 2); and others.

**Fig 2:**
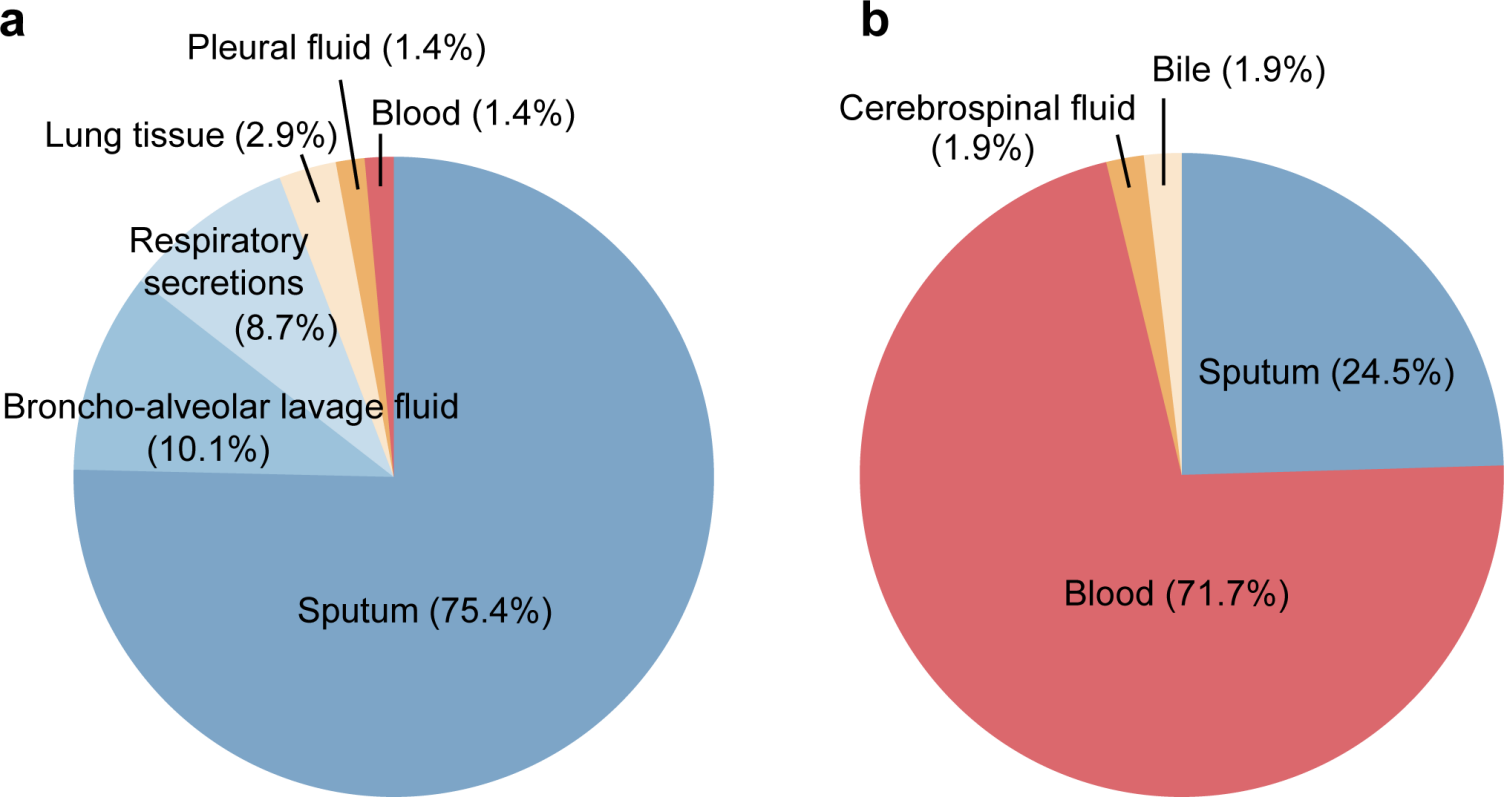
Distribution of isolated samples for different fungi in IFI group. (**a**) Distribution of sample sources for molds (*n* = 69); (**b**) Distribution of sample sources for yeasts (*n* = 53). IFI, invasive fungal infections.

The pathogen culture samples from all patients primarily consisted of three types: blood, respiratory samples (such as sputum and BALF), and sterile body fluids (Table S2). According to the culture results of the IFI group, molds (*n* = 69) were mainly isolated from respiratory samples (97.1%), while yeasts (*n* = 53) were predominantly derived from blood samples (71.7%) and partially from sputum (24.5%) (Fig. 2).

### Performance of BDG test and GM test for detection of IFI

In this study, BDG test showed a sensitivity of 64.6% (95% CI: 55.6%–73.2%) and a specificity of 83.1% (95% CI: 76.7%–88.4%), while GM test showed a significantly lower sensitivity (47.5%, 95% CI: 38.4%–56.8%) (*P* = 0.008) and a significantly higher specificity (93.0%, 95% CI: 88.1%–96.3%) (*P* = 0.003). The performance of the BDG test and GM test for detection of IFI is presented in Table 3.

**TABLE 3:** Specificity, sensitivity, positive predictive values, and negative predictive values of different tests for different species of pathogens^*a*^^,^^*b*^

Parameters	Sensitivity (%)	Specificity (%)	PPV (%)	NPV (%)	*P* value of sensitivity	*P* value of specificity
BDG test	64.6 (55.6–73.2)	83.1 (76.7–88.4)	73.1 (65.6–79.6)	76.9 (72.1–81.0)	0.008	0.003
GM test	47.5 (38.4–56.8)	93.0 (88.1–96.3)	82.9 (73.1–89.6)	71.4 (67.8–74.8)		
BDG test and GM test	32.8 (24.6–41.9)	97.1 (93.3–99.0)	88.9 (76.5–95.2)	67.1 (64.2–69.8)	<0.001	<0.001
BDG test or GM test	79.5 (71.3–86.3)	79.1 (72.2–84.9)	72.9 (66.5–78.5)	84.5 (79.2–88.6)		
BDG test for *Aspergillus* (*n* = 55)	61.8 (47.7–74.6)	100.0 (39.8–100.0)	100.0	16.0 (12.0–21.0)	0.832	NA
GM test for *Aspergillus* (*n* = 55)	65.5 (51.4–77.8)	100.0 (39.8–100.0)	100.0	17.4 (12.8–23.2)		
BDG test for *Candida* (*n* = 49)	67.3 (52.5–80.0)	82.5 (75.7–88.0)	54.1 (44.4–63.5)	89.2 (84.6–92.5)	<0.001	0.001
GM test for *Candida* (*n* = 49)	30.6 (18.3–45.4)	93.8 (88.8–97.0)	60.0 (41.9–75.7)	81.5 (78.5–84.2)		

^*a*^ BDG test, 1,3-β-D-glucan test; GM test, galactomannan antigen test; PPV, positive predictive value; NPV, negative predictive value.

^*b*^ NA, the value cannot be calculated.

**Fig 3:**
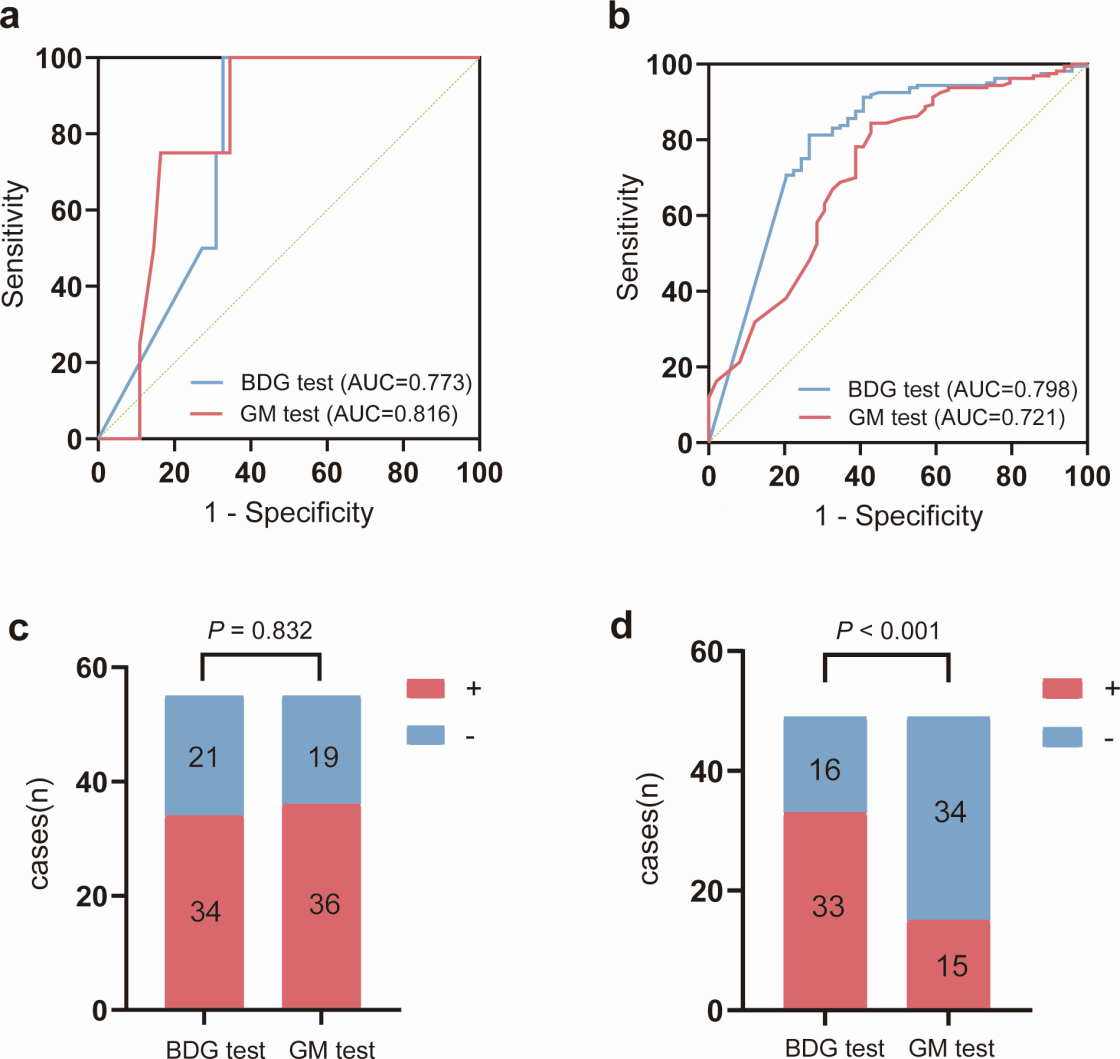
ROC curves and positive amount of BDG test and GM test for *Aspergillus* and *Candida* infections. This analysis includes all patients diagnosed with *Aspergillus* or *Candida* infections. (**a**) ROC curves comparing BDG and GM tests in patients with *Aspergillus* infection (AUC = 0.773 for BDG, 0.816 for GM); (**b**) ROC curves comparing BDG and GM tests in patients with *Candida* infection (AUC = 0.798 for BDG, 0.721 for GM); (**c**) distribution of BDG and GM test results (positive/negative) in *Aspergillus* infection; (**d**) distribution of BDG and GM test results in *Candida* infection. *P*-values are shown for comparisons between the two tests. ROC, receiver operating characteristic; AUC, the area under the curve; BDG test, 1,3-β-D-glucan test; GM test, galactomannan antigen test. “+” indicates positive results; “–” indicates negative results.

Compared to CRS, ROC curve analysis showed that GM test (AUC = 0.816, 95% CI: 0.694–0.905) was similar to BDG test (AUC = 0.773, 95% CI: 0.645–0.872) in the detection of IA (*P* = 0.696, *Z* test) (Fig. 3a). Consistently, for the 55 IA patients, there was no statistically difference between BDG test (61.8%, 34/55) and GM test (65.5%, 36/55) in terms of positive rates (*P* = 0.832) (Fig. 3c). The performance of the BDG test (AUC = 0.798, 95% CI: 0.737–0.850) was better than GM test (AUC = 0.721, 95% CI: 0.655–0.781) in the detection of IC (*P* = 0.123, *Z* test) (Fig. 3b). Notably, for the 49 IC patients, the positive rate of BDG test (67.3%, 33/49) was significantly higher than that of GM test (30.6%, 15/49) (*P* < 0.001) (Fig. 3d). In addition, the BDG test and GM test showed no difference in results across different species of *Aspergillus* and *Candida* (Tables S3 and S4).

### Performance of prediction diagnostic probability value

**Fig 4:**
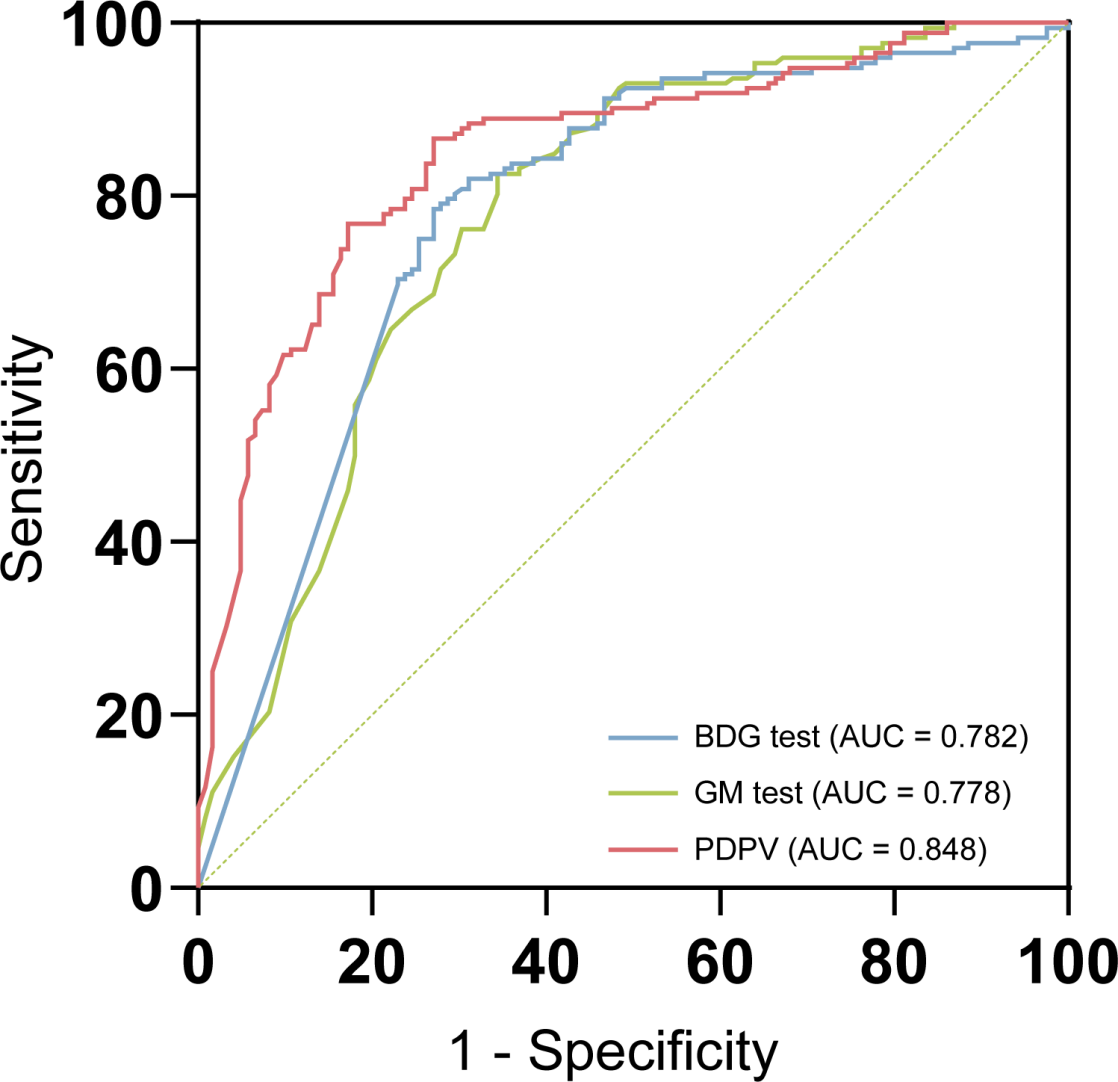
Comparison of ROC curves for different tests. The BDG test, GM test, and PDPV (predicted diagnostic probability value derived from combined diagnostic models) are shown. AUC was 0.782 for the BDG test, 0.778 for the GM test, and 0.848 for the PDPV model, indicating improved performance of the combined approach. ROC, receiver operating characteristic; AUC, the area under the curve; BDG test, 1,3-β-D-glucan test; GM test, galactomannan antigen test.

According to the ROC curve (Fig. 4; Table S5), AUC values for the BDG test and GM test were 0.782 (95% CI: 0.730–0.828) and 0.778 (95% CI: 0.726–0.824), respectively (*P* = 0.905, *Z* test). Then, the thresholds of BDG and GM tests were redefined based on the data from this study. ROC curves were constructed, and the optimal cut-off values were determined at the maximum Youden index point (BDG test: 37.31 pg/mL; GM test: 0.30). These new thresholds demonstrated higher sensitivity, providing a potential reference for threshold adjustment (Table S6).

Logistic regression analysis demonstrated that both BDG and GM test values were independent predictors of IFI (*P* < 0.001). The regression equation was as follows: PDPV = logit(*P*) = −1.399 + 1.375 × GM value + 0.005 × BDG value, PDPV, the predicted probability derived from this model, served as a composite diagnostic index. ROC curve analysis based on PDPV values showed an AUC of 0.848 (95% CI: 0.802–0.887) (Table S5). The optimal threshold, determined by the maximum Youden index (*J* = 0.596), was 0.64, corresponding to a sensitivity of 73.0% and a specificity of 86.6%. Comparative ROC analysis demonstrated that PDPV significantly outperformed both the BDG test (*P* = 0.010) and the GM test (*P* = 0.002) in detecting IFI (*Z* test) (Table 4).

**TABLE 4:** Pairwise comparison of ROC curves^*b*^

Parameters	BDG test ~GM test	BDG test ~PDPV	GM test ~PDPV
*Z* value**^*a*^**	0.119	3.297	3.063
*P* value	0.905	0.010	0.002

^*a*^ The test method is DeLong’s test.

^*b*^ BDG test, 1,3-β-D-glucan test; GM test, galactomannan antigen test; PDPV, predicted diagnostic probability value.

To assess the internal robustness of the model, a bootstrap validation procedure with 1,000 resamples was performed in R. The mean AUC across bootstrap samples was 0.848, with a 95% bootstrap confidence interval of 0.799 to 0.893, confirming the internal stability and discriminatory capacity of the PDPV model (Fig. S1).

## DISCUSSION

The retrospective study analyzed IFI in patients with hematologic diseases undergoing HSCT, confirmed the presence of IFI using culturing techniques and integrating existing diagnostic criteria, and evaluated the sensitivity and specificity of the BDG and GM tests in diagnosing different types of IFI.

It was found that the main pathogens infecting IFI patients were *Aspergillus* and *Candida* species, which are the two most common pathogens responsible for invasive infections, particularly in immunocompromised patients. These pathogens are also considered the leading causes of IFI in hematological malignancy patients in Asia (14). Research in several European countries has indicated that invasive aspergillosis predominates among IFD patients, followed by invasive candidiasis and Pneumocystis pneumonia (15). Similarly, studies from the Americas also report that invasive aspergillosis is the primary IFD, followed by candidemia and zygomycosis (16). These pathogen compositions are generally consistent with those identified in this study.

In clinical practice, isolating *Aspergillus* from human specimens strongly indicates a fungal infection, as it is not part of the normal microbiota and requires immediate attention. However, *Aspergillus* may occasionally be isolated as environmental contaminants, so the interpretation of culture results should be made in conjunction with clinical context and additional mycological evidence to ensure diagnostic accuracy. In contrast, *Candida* is a natural component of the respiratory microbiota, so its isolation from respiratory samples, like sputum or nasal secretions, doesn’t necessarily confirm an infection. In such cases, it is important to combine other clinical indicators to confirm IFI.

It is widely acknowledged that galactomannan, an integral component of the cell walls in *Aspergillus*, is one of the initial antigens detectable within the bloodstream or other bodily secretions. Consequently, the GM test plays an important role in the diagnosis of IA. It should be noted that the GM test may cross-react with *Cryptococcus*, *Penicillium*, *Paecilomyces*, *Histoplasma*, etc., potentially producing false-positive results in non-*Aspergillus* cases (17–19). In contrast, BDG is a polysaccharide found in the cell walls of various fungal species, such as *Candida*, *Aspergillus*, *Penicillium*, *Fusarium,* and *Pneumocystis jirovecii* (20–22) which is released into the bloodstream and bodily fluids in increased amounts after being engulfed by human immune cells. Nevertheless, the BDG test (which non-specifically detects IA) and the GM test have shown similar sensitivity in the diagnosis of IA (Table 3). While the BDG test demonstrated significantly higher sensitivity than the GM test in detecting IC, though with lower specificity, it showed promising diagnostic efficacy for IC. These results were similar to previous studies (11, 22, 23). Notably, in addition to pathogen-related cross-reactivity, both assays may be affected by non-fungal factors (24, 25) such as intravenous certain β-lactam antibiotics (e.g., piperacillin–tazobactam), intravenous immunoglobulin (IVIG) administration, and blood transfusions, which may further reduce their specificity.

Furthermore, it should be recognized that after receiving antifungal prophylaxis or treatment, such as azoles, echinocandins, or amphotericin B, the positive rate, sensitivity, and auxiliary diagnostic value of the BDG test or GM test will decrease (26–28). In this study, the proportion of patients in the IFI group who received one or more antifungal drugs was significantly higher than that in the non-IFI group (Table S1). This difference may have led to suppressed biomarker levels in some IFI patients, thereby underestimating the sensitivity of the BDG and GM tests.

When *Aspergillus* and *Candida* infections were analyzed separately in the IFI group, it was observed that both BDG test and GM test had low sensitivity but high specificity in diagnosing *Aspergillus* infections. Studies have shown that the impact of immunosuppression on BDG or GM levels during IA infections has not been well quantified (29). As the subjects of this study were patients with hematologic diseases undergoing chemotherapy or HSCT and were in an immunosuppressed state, BDG and GM levels in serum may be influenced by host immunosuppression. Additionally, since only cases with positive culture results were included in the study, negative cases of *Aspergillus* infections were inadvertently excluded. This resulted in only four patients having positive culture results but not being diagnosed with IA, which may overestimate the specificity of the GM test.

For infections caused by different *Aspergillus* species, there were no significant statistical differences in the performance between the BDG and GM tests, which aligns with the research by Magira et al. (30). However, some studies have suggested that the BDG and GM tests may perform differently in diagnosing *Aspergillus* species, with lower sensitivity for detecting *A. fumigatus* compared to other species, and that the BDG test may perform better than the GM test (11). As for *Candida* infections, there are no detailed studies comparing BDG and GM tests for different species. In the analysis, no significant difference was found in BDG and GM test results for different *Candida* infections.

This study also explored the potential benefits of combining the BDG and GM tests for detecting IFI. The diagnostic thresholds for both tests were reassessed. ROC curve analysis determined optimal cut-off values of 37.31 pg/mL for the BDG test and 0.30 for the GM test. These revised thresholds enhanced sensitivity, providing a more accurate reference for IFI detection (Table S6). Furthermore, both parallel (either test positive) and serial (both tests positive) combinations of BDG and GM were explored and improved the sensitivity and specificity to 79.5% and 97.1%, respectively (Table 3). Previous studies on invasive aspergillosis used similar combination strategies, and these findings underscore the value of traditional binary combinations (30, 31). However, this study innovatively extends this approach by developing a logistic regression model that integrates both markers into a continuous risk score, which may offer better diagnostic precision and adaptability for broader IFI detection. The new indicator “PDPV”, which is generated by a binary regression model, showed improved diagnostic performance, and ROC analysis confirmed that PDPV outperformed both the BDG and GM tests individually. This supports the advantage of using combined biomarkers for IFI detection. The combined model has the potential to reduce the risk of misdiagnosis and may serve as a useful diagnostic tool pending further validation in clinical practice. These findings should be validated in larger studies to confirm their generalizability.

What’s more, the study also identified risk factors for IFI occurrence and found significant differences between the IFI group and non-IFI group in terms of HSCT, history of fungal and bacterial infections, respiratory diseases, gastrointestinal mucosal injury, liver dysfunction, etc. These results were consistent with previous studies (3, 4, 31, 32).

### Conclusions and limitations

In conclusion, the composite diagnostic indicator PDPV in the study significantly improved the diagnostic performance for IFI, achieving higher overall accuracy (AUC), increased sensitivity, and robust specificity compared to individual BDG or GM tests. Moreover, the BDG test showed a higher positive rate and diagnostic efficiency than the GM test in detecting IC, but the difference between the two methods was not significant in detecting IA.

While this study has several limitations. First, it was a single-center retrospective study and thus exploratory in nature, which may limit the generalizability of the results. However, Peking University People’s Hospital is a leading institution with a broad national reach, providing a representative sample and ensuring data consistency and reliability. Second, the analysis relied on a single serum sample for BDG test and GM test results. While two consecutive samples would improve diagnostic accuracy, a single measurement is more efficient, particularly in institutions with limited resources. These tests can serve as effective initial screening tools, especially for high-risk populations, though further investigation is needed for definitive diagnosis. Additionally, only serum GM values were used to assess IA, without incorporating BALF test. This may contribute to the low sensitivity observed. Finally, antifungal prophylaxis could influence diagnostic outcomes, as it may reduce test sensitivity. This should be considered when interpreting the results.

Despite these limitations, the evaluation, comparison, and combined use of the two detection methods, as well as the innovative application of the composite diagnostic indicator PDPV, provide valuable references for future multicenter studies in China or internationally.

## Data Availability

The data that support the findings of this study are available on request from the corresponding author. The data are not publicly available due to privacy or ethical restrictions.

## SUPPLEMENTAL MATERIAL

Supplemental Material spectrum.01209-25-s0001.docx Supplemental results, Tables S1 to S6, and Fig. S1.

ASM does not own the copyrights to Supplemental Material that may be linked to, or accessed through, an article. The authors have granted ASM a non-exclusive, world-wide license to publish the Supplemental Material files. Please contact the corresponding author directly for reuse.
