# Supplementary material for: Assessment of the 1,3-β-D-glucan test and the galactomannan antigen test in the detection of invasive fungal infections in patients with hematological diseases
Source: Microbiol Spectr. 2025 Sep 3;13(10):e01209-25. doi: 10.1128/spectrum.01209-25 (PMC12502599; doi:10.1128/spectrum.01209-25)
Supplement: Supplemental Material — Supplemental results, Tables S1 to S6, and Fig. S1. [file spectrum.01209-25-s0001.docx]

**Supplementary Material**

**Detailed Clinical Characteristics**

Among the 122 patients in the IFI group, the mean age was 47.0 ± 1.5 years, with 61.5% being male. In the 172 non-IFI patients, the mean age was 56.1 ± 1.3 years, with 60.5% male.

The distribution of underlying hematologic malignancies differed between groups. In the IFI group, acute myeloid leukemia (AML) was the most frequent diagnosis (45.1%, 55/122), followed by acute lymphoblastic leukemia (ALL, 14.8%, 18/122), and myelodysplastic syndromes (MDS, 9.8%, 12/122). In the non-IFI group, AML also predominated (37.2%, 64/172), but there was a notably higher proportion of multiple myeloma (MM, 22.7%, 39/172) and non-Hodgkin lymphoma (NHL, 15.1%, 26/172). Additional clinical characteristics, including selected comorbidities not discussed in the main text, were also assessed. No significant differences were observed between IFI and non-IFI patients in the prevalence of diabetes, renal insufficiency, or viral infections (*P* > 0.05 for all; see Table 1).

**Additional Fungal Species and Sample Sources**

Among the *Aspergillus* isolates (n = 55), non-*fumigatus Aspergillus* species accounted for 36 cases (65.5%). These included *Aspergillus flavus* (n = 8, 14.5%), *Aspergillus niger* (n = 6, 10.9%), other species (n = 6, 10.9%) such as *Aspergillus versicolor* and *Aspergillus terreus*, and *Aspergillus*-associated co-infections with multiple species or other molds (n=15, 27.3%). *Aspergillus fumigatus* alone was isolated in 19 cases (34.5%) and 1 (1.8%) case for co-infection of *Aspergillus fumigatus*.

Similarly, among the *Candida* isolates (n = 49), non-*albicans Candida* species accounted for 39 cases (79.6%). This included *Candida tropicalis* (n = 26, 53.1%), *Candida parapsilosis* (n = 8, 16.3%), *Candida glabrata* (n = 4, 8.2%), and co-infections involving non-*albicans* *Candida* (n = 1, 2.0%). In contrast, *Candida albicans* was identified in only 10 cases (20.4%).

In addition to common respiratory and bloodstream isolates, several fungal species were recovered from sterile body fluid specimens, indicating invasive disease. These included *Candida glabrata* from bile (n = 1), *Candida parapsilosis* from cerebrospinal fluid (n = 1), and *Mucor* from pleural effusion (n = 1). Although infrequent, these findings demonstrate the capacity of opportunistic fungi to disseminate and invade normally sterile anatomical sites in immunocompromised hosts.

**Fungal Isolates in Non-IFI Patients**

Among the non-IFI patients, fungal isolates were predominantly recovered from respiratory tract specimens, including sputum (n = 134), oral swabs (n = 18), throat swabs (n = 12), nasal swabs (n = 3), bronchoalveolar lavage fluid (n = 3), tracheal secretions (n = 1), while a single isolate was also obtained from gastric juice (n = 1). The most frequently identified species were *Candida albicans* (e.g., 68 cases in sputum, 15 in oral swabs), *Candida tropicalis*, *Candida glabrata*, and *Saccharomyces cerevisiae*. Other isolates included various non-*albicans Candida* species and filamentous fungi such as *Aspergillus fumigatus*, *Aspergillus niger*, and *Penicillium spp.*, all of which were judged to be colonizing or contaminants in the absence of clinical signs of infection.

**Table S1** Medication usage and mortality outcomes in IFI and non-IFI groups

| **Characteristics** | **IFI group**  **(n=122)** | **Non-IFI group**  **(n=172)** | ***P* value** | |
| --- | --- | --- | --- | --- |
| Medicate, n (%) |  |  |  | |
| Triazole | 116 (95.1) | 109 (63.4) | <0.001* | |
| Voriconazole | 96 (78.7) | 89 (51.7) | <0.001* | |
| Fluconazole | 18 (14.8) | 10 (5.8) | 0.010* | |
| Posaconazole | 51 (41.8) | 35 (20.3) | <0.001* | |
| Itraconazole | 12 (9.8) | 2 (1.2) | 0.029* | |
| Isavuconazole | 4 (3.3) | 0 (NA) |  | |
| Caspofungin | 88 (72.1) | 65 (37.8) | <0.001* | |
| Amphotericin B | 30 (24.6) | 16 (9.3) | <0.001* | |
| Natamycin | 27 (22.1) | 34 (19.8) | 0.622 | |
| Combination of drugs | 53 (43.4) | 56 (37.8) | 0.239 | |
| Prognosis, n (%) |  |  |  | |
| Death occurs within a year of infection | | 58 (47.5) | 52 (30.3) | 0.003* |
| Death occurs within 30 days of infection | 9 (7.4) | 16 (9.3) | 0.560 | |

**P*<0.05

IFI, Invasive Fungal Infections

**Table S2** Distribution of fungal species isolated from sterile body fluids

| Sample Type | Count | Fungal Species |
| --- | --- | --- |
| Blood | n = 39 | *Candida tropicalis* (n=24) |
|  |  | *Candida parapsilosis* (n=5) |
|  |  | *Candida albicans* (n=3) |
|  |  | *Trichosporon asahii* (n=2) |
|  |  | *Candida glabrata* (n=1) |
|  |  | *Saccharomyces japonicus* (n=1) |
|  |  | *Rhodotorula mucilaginosa* (n=1) |
|  |  | *Fusarium* (n=1) |
|  |  | *Candida parapsilosis + Fusarium* (n=1) |
| Bile | n = 1 | *Candida glabrata* |
| Cerebrospinal Fluid | n = 1 | *Candida parapsilosis* |
| Pleural Fluid | n = 1 | *Rhizopus* |

**Table S3** Distribution of BDG and GM test results among different types of *Aspergillus* Infections

| **BDG/GM test range values** | ***Aspergillus fumigatus***  **(n=19)** | ***Aspergillus flavus***  **(n=8)** | ***Aspergillus niger***  **(n=6)** | **Other *Aspergillus***  **(n=6)** | **Multi-*Aspergillus* infections**  **(n=16)** | ***P* values** |
| --- | --- | --- | --- | --- | --- | --- |
| BDG test negative  (0.0< BDG < 60.0 pg/ml) | 8 (42.1%) | 2 (25.0%) | 3 (50.0%) | 2 (33.3%) | 6 (37.5%) | 0.935 |
| BDG test positive  (≥ 60.0 pg/ml) | 11 (57.9%) | 6 (75.0%) | 3 (50.0%) | 4 (66.7%) | 10 (62.5%) |  |
| BDG test median  (range pg/ml) | 63.7  (10.00-564.26) | 175.41  (10.00-258.89) | 99.22  (10.00-171.69) | 184.93  (10.00-782.18) | 122.34  (10.00-546.10) | 0.666 |
| GM test negative  (0.0 < GM < 0.5) | 8 (42.1%) | 2 (25.0%) | 4 (66.7%) | 1 (16.7%) | 5 (31.3%) | 0.428 |
| GM test positive (≥ 0.5) | 11 (57.9%) | 6 (75.0%) | 2 (33.3%) | 5 (83.3%) | 11 (58.7%) |  |
| GM test median (range) | 0.50  (0.12-10.03) | 0.73  (0.11-7.54) | 0.34  (0.13-0.83) | 0.63  (0.26-6.20) | 2.84  (0.26-9.28) | 0.348 |

BDG test, 1,3-β-D-Glucan test; GM test, Galactomannan antigen test

**Table S4** Distribution of BDG and GM test results among different types of *Candida* Infections

| **BDG/GM test range values** | ***Candida albicans***  **(n=7)** | ***Candida tropicalis***  **(n=26)** | ***Candida parapsilosis***  **(n=8)** | ***Candida glabrata***  **(n=4)** | **Multi-*Candida* infections**  **(n=4)** | ***P* values** |
| --- | --- | --- | --- | --- | --- | --- |
| BDG test negative  (0.0< BDG < 60.0 pg/ml) | 2 (28.6%) | 10 (38.5%) | 4 (50.0%) | 0 (0) | 0 (0) | 0.328 |
| BDG test positive  (≥ 60.0 pg/ml) | 5 (71.4%) | 16 (61.5%) | 4 (50.0%) | 4 (100%) | 4 (100%) |  |
| BDG test median  (range pg/ml) | 167.73  (10.00-808.56) | 127.75  (10.00-633.48) | 106.90  (10.00-768.76) | 107.76  (66.40-186.94) | 174.11  (97.07-290.00) | 0.939 |
| GM test negative  (0.0 < GM < 0.5) | 3 (42.9%) | 20 (76.9%) | 6 (75.0%) | 2 (50.0%) | 2 (50.0%) | 0.330 |
| GM test positive (≥ 0.5) | 4 (57.1%) | 6 (23.1%) | 2 (25.0%) | 2 (50.0%) | 2 (50.0%) |  |
| GM test median (range) | 0.49  (0.16-1.76) | 0.30  (0.14-3.74) | 0.29  (0.16-0.75) | 0.49  (0.37-0.87) | 1.22  (0.13-5.63) | 0.125 |

BDG test, 1,3-β-D-Glucan test; GM test, Galactomannan antigen test

**Table S5** Comparison of AUC values for BDG test, GM test, and PDPV in IFI diagnosis

| **Variable** | **AUC** | **SE** | **95% CI** |
| --- | --- | --- | --- |
| BDG test | 0.782 | 0.027 | 0.730-0.828 |
| GM test | 0.778 | 0.029 | 0.726-0.824 |
| PDPV | 0.848 | 0.023 | 0.802-0.887 |

BDG test, 1,3-β-D-Glucan test; GM test, Galactomannan antigen test; PDPV, predicted diagnostic probability value; IFI, Invasive Fungal Infections; AUC: Area under the curve; SE: Standard Error; 95% CI: 95% Confidence Interval

**Table S6** Diagnostic Performance between BDG test and GM test at the optimal cut-off values

| **Parameters** | **Sensitivity (%)** | **Specificity (%)** | **PPV (%)** | **NPV (%)** | **Youden index** |
| --- | --- | --- | --- | --- | --- |
| At optimal cut-off values: BDG test, 37.31 pg/mL; GM test, 0.30 | | | | | |
| BDG test | 72.9 | 78.5 | 70.6 | 80.4 | 0.514 |
| GM test | 65.6 | 82.6 | 72.7 | 77.2 | 0.481 |
| At manufacturer-provided cut-off values: BDG test, 60 pg/mL; GM test, 0.50 | | | | | |
| BDG test | 64.6 | 83.1 | 73.1 | 76.9 | 0.477 |
| GM test | 47.5 | 93.0 | 82.9 | 71.4 | 0.405 |

BDG test, 1,3-β-D-Glucan test; GM test, Galactomannan antigen test; PPV: Positive Predictive Value; NPV: Negative Predictive Value


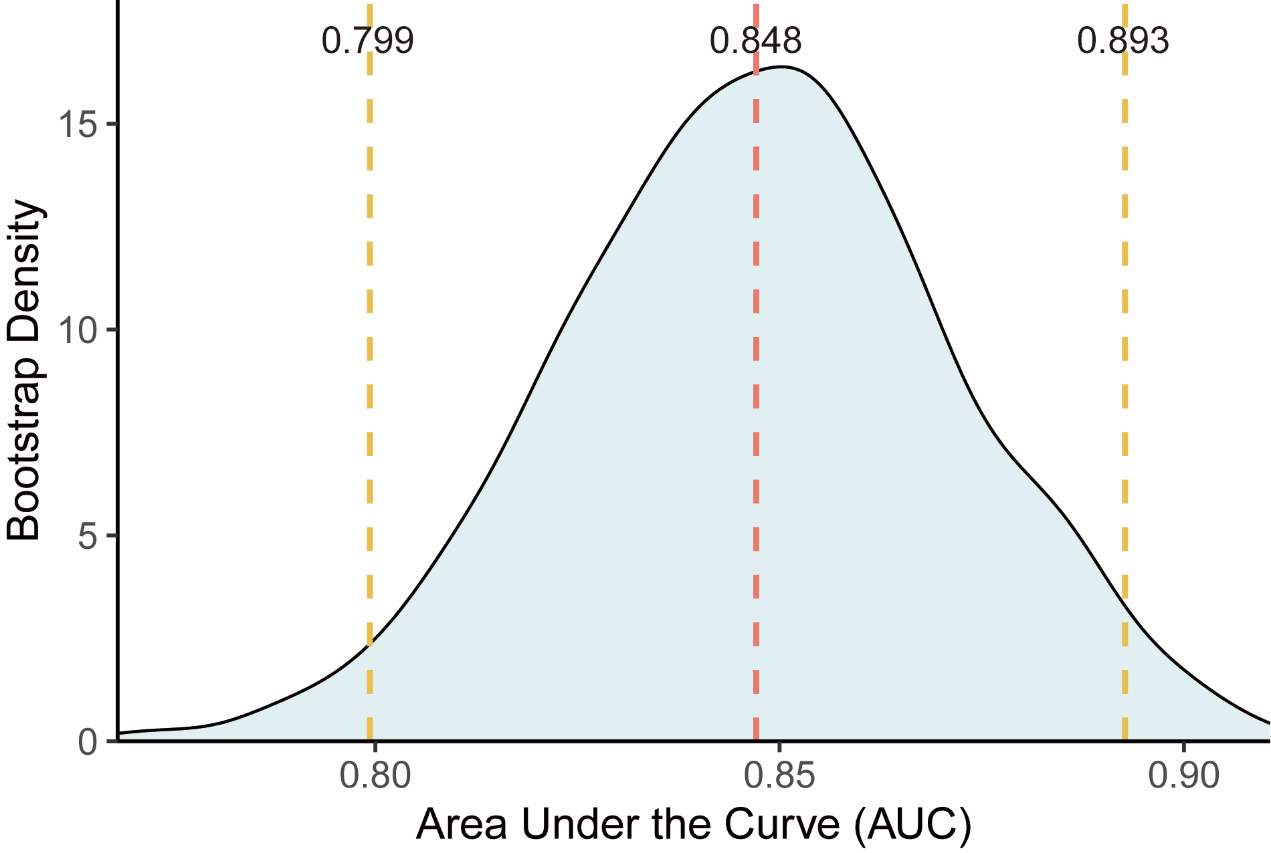


**Figure S1**. Bootstrap distribution of AUC values for internal validation of the PDPV model.

A kernel density plot was generated based on 1,000 bootstrap resamples to assess the internal stability and discriminative performance of the model. The x-axis represents the area under the ROC curve (AUC), while the y-axis denotes the unitless probability density (the total area under the curve equals 1). The dashed red vertical line indicates the mean AUC (0.848), and the dashed yellow lines represent the 95% bootstrap confidence interval (0.799 to 0.893). The distribution demonstrates a high concentration of AUC values in the upper range, supporting the robustness and generalizability of the PDPV model.

ROC: receiver operating characteristic; PDPV, predicted diagnostic probability value
